# Multifunctional Peptides from Equine Milk Lactoferrin: Evaluation of Antimicrobial Activity In Silico and In Vitro

**DOI:** 10.3390/ani16081223

**Published:** 2026-04-16

**Authors:** Meiramkul Narmuratova, Shara Atambayeva, Gulzhan Kaiyrmanova, Saltanat Orazova, Gulzhan Narmuratova, Bernard Faye

**Affiliations:** 1 Department of Biotechnology, Faculty of Biology and Biotechnology, Al-Farabi Kazakh National University, Al-Farabi Ave. 71/19, Almaty 050040, Kazakhstan; narmuratova@kaznu.kz (M.N.); atambayevashara@gmail.com (S.A.); saltanat.orazova@kaznu.edu.kz (S.O.); narmuratova15@gmail.com (G.N.); 2 Department of Biology, Institute of Natural Sciences, Kazakh National Women’s Teacher Training University, Aiteke Bi Street 99, Almaty 050000, Kazakhstan; 3 Centre de Coopération Internationale en Recherche Agronomique pour le Développement (CIRAD), Département Environnement et Sociétés, UMR-Systèmes d’Elevage en Milieux MEditerranéens et Tropicaux (SELMET), Campus International de Baillarguet, 34398 Montpellier, France

**Keywords:** equine milk lactoferrin, peptides, in silico prediction, in vitro validation, *Escherichia coli*, *Staphylococcus aureus*, *Pseudomonas aeruginosa*, antimicrobial

## Abstract

The rapid global spread of antimicrobial resistance among pathogenic microorganisms poses a serious challenge to both human and animal health, underscoring the urgent need for new strategies to combat resistance. Antimicrobial peptides (AMPs), key components of the innate immune system, are promising candidates because they disrupt the membranes of bacteria, fungi, and viruses, thereby reducing the risk of resistance development. Lactoferrin (LF), a multifunctional iron-binding glycoprotein abundant in mammalian milk, is a rich source of AMPs. Cationic peptide fragments such as lactoferricin and lactoferrampin exhibit more potent direct antimicrobial activity than the intact protein. Our previous studies have shown that peptides derived from Equine milk lactoferrin exhibit antihypertensive, anti-inflammatory, and anti-oncogenic activity in silico, highlighting their multifunctional bioactive potential. Building on these results, the present study aims to investigate the antimicrobial properties of these peptides. We used an integrated approach combining computer modeling and in vitro studies to identify and validate novel antimicrobial peptides from equine milk lactoferrin. Bioinformatics tools, including AMPScanner and CAMP, were used to predict antimicrobial domains, followed by experimental testing against *Escherichia coli*, *Staphylococcus aureus*, and *Pseudomonas aeruginosa*. The results showed that equine milk lactoferrin peptides possess potent and selective antimicrobial activity, with efficacy varying across bacterial species. These data expand the functional profile of lactoferrin-derived peptides, demonstrating their multifunctionality, and suggest that equine milk lactoferrin represents a promising natural source of antimicrobial agents, supporting alternative strategies to reduce antibiotic use in human and veterinary medicine.

## Simple Summary

Antibiotic resistance is becoming a serious problem in both medicine and animal production, so new natural substances that can fight harmful bacteria are urgently needed. In this study, we examined lactoferrin, a natural protein found in equine milk, as a possible source of antibacterial compounds. Using computer-based methods, we first identified small protein fragments likely to exhibit antimicrobial activity. We then tested lactoferrin and its breakdown products in the laboratory against several important bacteria. The strongest antibacterial effect was observed after 4 h of enzymatic treatment, suggesting that active fragments were released from the protein during this process. These fragments inhibited the growth of various bacterial strains. Our findings show that equine milk lactoferrin may be a promising natural source of new antibacterial substances and could contribute to the development of alternative approaches that help reduce antibiotic use.

## 1. Introduction

The global growth of antimicrobial resistance (AMR) represents one of the most serious threats to modern healthcare. According to the data of systemic analysis, bacterial AMR directly caused about 1.27 million deaths in 2019 and was associated with nearly 5 million fatal outcomes [1,2]. This problem is further aggravated by the excessive and often inappropriate use of antibiotics in human and veterinary medicine, as well as in agriculture [3].

Under conditions of a limited influx of new antibiotic-resistant cases, particular attention is being paid to identifying alternative antimicrobial agents with a low potential for resistance development. Antimicrobial peptides of the innate immune system, which act by disrupting microbial membrane integrity, are considered promising candidates due to their multiple targets and the slow emergence of resistance [4].

Among the natural sources of AMPs is lactoferrin (LF), a multifunctional iron-binding glycoprotein of the transferrin family that is present in mammalian milk, saliva, tears, and neutrophilic granules [5,6]. The primary contributors to its antimicrobial activity are cationic peptide fragments generated during proteolytic cleavage, particularly lactoferricin (Lfcin) and lactoferrampin, which originate from the N-terminal domain of the protein [6].

Most studies have focused on lactoferrin of bovine and human origin. However, lactoferrin from equine milk (eLF) remains insufficiently studied despite its unique composition [7,8]. Equine milk belongs to the “albumin” type (similar to human milk) and contains high levels of whey proteins, including lactoferrin and lysozyme. The concentrations of these proteins are significantly higher than in cow’s milk [9]. These components exhibit a pronounced synergy in their antimicrobial and immunomodulatory effects [10]. Recent studies confirm that equine milk and eLF hydrolysates generate bioactive peptides with antibacterial, antiviral, antihypertensive, anti-inflammatory, and antiangiogenic properties [11]. Equine milk and its fermented products (such as qymyz) are considered functional foods with a high content of protective factors that help maintain the microbiome and innate immunity [10]. In central Asia, including Kazakhstan, equine milk and qymyz are traditionally consumed [12], and antimicrobial peptides derived from equine lactoferrin may help maintain animal health and reduce the use of antibiotics in livestock farming. Despite the progress, specific cryptic antimicrobial sequences in the structure of lactoferrin from equine milk remain insufficiently studied. Conventional methods for isolating and testing peptides are labor-intensive and costly. Modern bioinformatics tools and machine learning algorithms (including deep neural networks) enable high precision in silico screening of potential AMPs, reducing false positives and accelerating the selection of candidates for experimental validation [13,14]. Despite existing data on the antimicrobial activity of equine lactoferrin hydrolysates, specific hidden peptide sequences responsible for this effect remain poorly characterized experimentally. The present study aims to identify new antimicrobial peptides within the structure of equine milk lactoferrin using a consensus bioinformatic approach. The findings may facilitate the development of innovative agents to combat resistant infections [11,15,16].

## 2. Materials and Methods

### 2.1. In Silico Identification of Antimicrobial Peptides

#### 2.1.1. Study Objects

Peptide sequences were identified by liquid chromatography coupled with tandem mass spectrometry (LC-MS/MS) using an Impact II ESI-QTOF mass spectrometer (Bruker Daltonics, Mannheim, Germany) coupled to a Dionex UltiMate 3000 nano-HPLC system (Thermo Fisher Scientific, Waltham, MA, USA) [17]. Peptides were separated on an Acclaim PepMap RSLC C18 column with a 75 min acetonitrile gradient(Thermo Fisher Scientific, USA). Data were processed with DataAnalysis 3.4 and searched using Mascot 2.6.1 against the Swiss-Prot database (release 2024_02; taxonomy: Other mammals and *Equus caballus* lactoferrin), specifying trypsin digestion, carbamidomethylation of cysteine, and oxidation of methionine. Mass tolerances were 100 ppm for precursor ions and 0.05 Da for fragment ions. A total sequence coverage of 78% for lactoferrin was achieved, with 56 unique peptides identified for subsequent in silico antimicrobial prediction. (Supplementary Table S1).

#### 2.1.2. Screening Databases and Assessment of Novelty

For verification of the novelty of the obtained peptides and to exclude previously described molecules, a screening was carried out using curated international databases of antimicrobial peptides:

An exact-match search was performed among natural and synthetic AMPs using the APD3 (Antimicrobial Peptide Database; web server; accessed in January 2026). Screening for the presence of homological sequences among peptides with confirmed activity. The absence of exact matches in these data repositories was considered evidence of the scientific novelty of the identified sequences (DBAASP v3—Database of Antimicrobial Activity and Structure of Peptides; web server, accessed in March 2026) [18]. Additionally, a similarity analysis using NCBI BLASTp web server (accessed in March 2026) was performed for the lead candidates (SEQ14, SEQ48, and SEQ4) against the NCBI non-redundant protein database and APD3 to further evaluate their similarity to known antimicrobial peptides. BLASTp searches were conducted using the PAM30 matrix and a word size of 2. In this study, sequence-level novelty was defined primarily by the absence of identical matches in APD3 and DBAASP, while BLASTp was used as a supportive similarity-screening tool; candidates were considered potentially novel when no high-similarity matches (>80%) to previously reported independent antimicrobial peptides were detected.

#### 2.1.3. Analysis of Physicochemical Properties

Physicochemical parameters were calculated, and in silico prediction of antimicrobial potential was performed for each identified peptide. Using bioinformatics algorithms from the Antimicrobial Peptide Database (APD), the following parameters were determined: probability of biological activity (ScoreBioact), molecular weight (MS), isoelectric point (pI), aliphatic index, average hydropathy (GRAVY), and the Boman index to assess the ability to bind to proteins.

#### 2.1.4. Prediction of Antimicrobial Activity

To predict biological activity, a comprehensive approach combining multiple machine learning algorithms was used. Three classifiers were applied for cross-validation: the Support Vector Machine (SVM), the Random Forest method (RF), and Artificial Neural Networks (ANN) methods [13,19]. A probability threshold value of ≥0.5 was used to classify a peptide as an AMP.

To increase prediction accuracy, we used an additional deep learning model (AMPScanner v2), which is characterized by high sensitivity to novel, previously undescribed sequences [20]. The earlier version of the algorithm, AMPScanner vr.1, was also used as an auxiliary tool. The threshold of ≥0.5 for CAMPR3 classifiers (RF, SVM, ANN) was selected as a standard binary classification cutoff to maintain high sensitivity during initial screening. In contrast, a more stringent threshold of ≥0.6 was applied for AMPScanner v2 to prioritize sequences with the highest structural probability of activity, thereby reducing the risk of false positives inherent in deep learning models when processing novel, cryptic sequences.

#### 2.1.5. Candidate Eligibility Criteria

The selection of the most promising peptides for subsequent synthesis was carried out based on a rating system. The following sequences were considered a priority if they met these criteria: a positive prediction in the AMPScanner v2 model (≥0.6); confirmation of antimicrobial activity in the CAMP classifier (primarily according to the Random Forest algorithm); the presence of structural motifs characteristic of known antimicrobial peptides (amphiphilicity, the presence of cationic amino acid residues).

### 2.2. In Vitro Evaluation of Antimicrobial Activity

#### 2.2.1. Obtaining Lactoferrin and Its Enzymatic Hydrolysates

Native lactoferrin from equine milk was used in the study. Enzymatic hydrolysis was carried out using trypsin to obtain peptide fragments, and the reaction was conducted for 2, 3, 4, and 8 h. After completion of the enzymatic treatment, the samples were used to assess antimicrobial activity. In the antimicrobial assay, native lactoferrin and its hydrolysates were tested at a concentration of 20 mg/mL.

#### 2.2.2. Microbial Test Cultures

Antimicrobial activity was evaluated against clinically significant bacterial species. In this study, *Escherichia coli*, *Staphylococcus aureus*, and *Pseudomonas aeruginosa*, obtained from a laboratory microbiology collection, were used as test cultures. These species are widely known and commonly used as standard reference organisms for assessing antimicrobial activity. Although the strains are not associated with international culture collections (e.g., ATCC), they were maintained under controlled laboratory conditions and consistently demonstrated stable morphological, cultural, and biochemical characteristics typical of each species, ensuring their suitability as representative test organisms. Strain identification was based on morphological, cultural, and biochemical characteristics using standard microbiological methods. The observed characteristics were fully consistent with established descriptions for these species.

#### 2.2.3. Determination of Antimicrobial Activity

Antimicrobial activity of samples was determined using the agar well diffusion method on Mueller–Hinton agar. Briefly, each strain was grown overnight in nutrient broth at 37 °C, and the bacterial suspension was adjusted to a 0.5 McFarland standard (≈1.5 × 10^8^ CFU/mL) using sterile saline and spectrophotometric measurement. A 100 µL aliquot of the standardized suspension was spread uniformly onto the surface of Mueller–Hinton agar plates using a sterile spreader. After allowing the inoculum to absorb at room temperature for 10–15 min, wells of 6 mm diameter were aseptically punched into the agar using a sterile cork borer. Each well was filled with 100 µL of the test sample (native lactoferrin or its hydrolysates) at the desired concentration. A sterile solvent (distilled water or appropriate buffer) served as the negative control, while ciprofloxacin (0.01 mg/mL) was used as the positive control. Native lactoferrin was included as a reference compound to allow comparative evaluation of the hydrolysates.

Plates were incubated at 37 °C for 24 h under aerobic conditions. All assays were performed in triplicate. Inhibition zone diameters were measured in millimeters and reported as mean ± standard deviation. Antimicrobial activity was assessed by comparing the inhibition zones of each sample with those of the positive control, negative control, and native lactoferrin, providing a clear comparative framework for evaluating relative efficacy.

### 2.3. Statistical Analysis of Data

Results of the antimicrobial assay were expressed as mean inhibition zone diameters (mm) based on three independent replicates. For the in vitro part of the study, differences between native lactoferrin and its hydrolysates obtained at different hydrolysis times were assessed separately for each bacterial strain using one-way analysis of variance (ANOVA), followed by Fisher’s least significant difference (LSD) test. For the in silico part of the study, peptide sequences were classified according to the predicted bioactivity score, and differences in physicochemical parameters between groups were also assessed using one-way ANOVA followed by Fisher’s LSD test. Statistical analyses were performed using XLSTAT version 2023.3.0.1415 (Addinsoft ©) in Microsoft Excel. Differences were considered statistically significant at *p* < 0.05

## 3. Results

### 3.1. Comprehensive In Silico Screening and Identification of Bioactive Peptides

At the initial screening stage, 56 lactoferrin-derived peptides identified by LC–MS/MS were evaluated in silico (Supplementary Table S2). The predicted bioactivity score (ScoreBioact) ranged from 0.06 to 0.95 across the dataset. Using the predefined threshold (ScoreBioact > 0.5), 23 peptides (41.1%) were classified as Class 1 (probable AMPs), while 33 peptides (58.9%) fell into Class 2 (inactive peptides). The average ScoreBioact was 0.647 for Class 1 compared to 0.262 for Class 2 (Supplementary Table S2).

To further prioritize synthesis candidates, a consensus ML/DL strategy was applied using CAMPR3 classifiers (RF/SVM/ANN; AMP threshold ≥0.5) and AMPScanner v2 (≥0.6), according to the predefined thresholds. This funnel reduced the candidate list to three priority peptides (5.4% of the initial set) that satisfied the ≥3/4 consensus requirement: SEQ14 (FCLFK) showed High Confidence (4/4) agreement, while SEQ48 and SEQ4 met the Moderate (3/4) consensus threshold (Table 1).

#### 3.1.1. Classification of Peptides and Analysis of Physicochemical Profile

At the first stage, the initial set of 56 peptide sequences was divided into two target classes: Class 1—probable AMPs, and Class 2—inactive peptides, based on the threshold value of the predicted bioactivity (ScoreBioact > 0.5). Differences in physicochemical properties between the two groups were evaluated as described in Section 2.3 (Supplementary Table S2, Table 2). The following parameters were calculated and analyzed in silico as dependent variables:

- Predicted evaluation of biological activity (ScoreBioact).
- Molecular mass (MS).
- Isoelectric point (pI).
- Thermal stability index (Aliphatic Index).
- Overall hydrophobicity index (GRAVY).
- Protein binding potential index (Boman Index).

**Table 2:** Comparative characteristics of the physicochemical properties of peptide clusters (ANOVA).

Parameters (Descriptors)	Class 1 (Active-like)	Class 2 (Inactive-like)	*p*-Value	Interpretation
ScoreBioact (Predicted)	0.518	0.369	0.024	Class 1 has a significantly higher bioactivity potential
GRAVY (Hydrophobicity)	0.592	−0.652	<0.0001	Critical difference: Class 1 hydrophobic, Class 2 hydrophilic
Boman Index (Binding)	0.392	2.212	<0.0001	Class 1 is specific to membranes; Class 2 tends to engage in non-specific interactions
Aliphatic Index	100.12	67.22	<0.0001	Class 1 peptides are more thermostable and richer in aliphatic side chains

The identified peptide classes differed significantly in physicochemical properties associated with membranotropic potential (Table 2).

In addition to being statistically significant, the differences between the two classes were also substantial in magnitude of effect (Δ, difference in means). Class 1 was much more hydrophobic than Class 2, as indicated by its higher GRAVY value (0.592 vs. −0.652; Δ = 1.244), consistent with a more membrane-interacting profile. The Boman index was substantially lower in Class 1 (0.392) compared with Class 2 (2.212; Δ = −1.820), suggesting a reduced propensity for nonspecific protein binding and a greater likelihood of membrane targeting. Likewise, the aliphatic index was higher in Class 1 (100.12) than in Class 2 (67.22; Δ = 32.90), reflecting a larger contribution of aliphatic side chains and potentially increased structural stability. Finally, ScoreBioact also differed between classes (0.518 vs. 0.369; Δ = 0.149) (Table 2).

Class 1 peptides were identified as a priority group. A key marker of their potential is a positive value of the hydrophobicity index (GRVY = 0.592), which is a necessary condition for the primary adsorption of peptide onto the hydrophobic surface of the bacterial membrane. In contrast, Class 2 peptides exhibited a pronounced hydrophilic character (−0.652), which significantly reduces the likelihood of their penetrating the lipid bilayer. Additionally, analysis of the Boman index showed that Class 1 peptides (0.39) have high specificity. Boman’s criteria, summarized in 2003, low index values (≤1.0) are typical of classical antimicrobial peptides that act through a membranolytic mechanism. In contrast, high values (≥2.0), observed in Class 2, are characteristic of peptides that perform signaling functions (such as hormones) or that are prone to undesirable protein–protein interactions, which may increase the risk of side effects.

#### 3.1.2. Comparative Analysis of Predicting Algorithms (ML vs. DL)

For predicting antimicrobial activity, a consensus strategy was used combining four algorithms: RF, SVM, and ANN from the CAMPR3 server, and the deep convolutional neural network AMPScanner v2. Results of comparative analysis (Table 1) revealed a significant divergence (discrepancy) between the predictions of the classical methods (SVM/ANN) and the modern deep learning model (AMPScanner).Across all 56 peptides, the classical CAMPR3 classifiers were generally more permissive than the deep learning filter AMPScanner v2. Using the predefined probability cutoffs (≥0.5 for RF/SVM/ANN and ≥0.6 for AMPScanner v2), the CAMPR3 models, particularly SVM/ANN, more frequently assigned high AMP probabilities, whereas AMPScanner v2 behaved as a stricter filter.

Applying these filters, we prioritized three peptides for synthesis that met the ≥3/4 consensus threshold: SEQ14 (High Confidence, 4/4) and SEQ4 and SEQ48 (Moderate, 3/4) (Table 1).

#### 3.1.3. Analysis of False-Positive Results

A phenomenon of “overestimation” of activity by classical algorithms (SVM and ANN) was observed for several peptides, such as SEQ17 and SEQ3. These peptides received extremely high probability scores in the SVM models (0.98 and 0.82, respectively), but were classified as inactive (“Non-AMP”) by the deep learning model AMPScanner v2, with probabilities close to zero (0.03 and 0.04).

This discrepancy is likely because classical models (SVM/ANN) rely heavily on amino acid composition (e.g., the presence of certain residues) but do not fully capture positional context. In contrast, convolutional networks (AMPScanner) analyze sequence patterns and can filter out peptides that formally have the “correct” composition but do not form an active structure. We therefore excluded SEQ17 and SEQ3 from the candidates, based on the stricter deep learning-based filter. The discrepancy underscores the limitations of individual in silico models, where classical algorithms (SVM, ANN) may overestimate activity, focusing on amino acid composition alone. By using a multi-model consensus, we mitigated this risk and prioritized only peptides with high structural confidence for further study.

#### 3.1.4. Identification of Lead Compound

The highest priority was assigned to peptides that demonstrated stable activity in all models (Consensus > 3/4) and belong to physicochemical Class 1. SEQ14 (FCLFK) was identified as the most promising predicted candidate from the screening. Consensus: all four algorithms recognized it as an AMP. The ANN model estimated the probability of its activity at 0.99, while AMPScanner estimated it at 0.86.

Importantly, the shortlisted lead peptides originate from the N-terminal N-lobe of equine lactoferrin, a region known to harbor cryptic cationic motifs released upon proteolysis. This aligns with our experimental finding that the increase in antimicrobial activity after trypsin hydrolysis is associated with the release of predicted AMP-like fragments located in the N-terminal region of equine lactoferrin. Notably, the best characterized lactoferrin-derived antimicrobial motifs (lactoferricins and related fragments, as well as lactoferampin) are also generated from the N-terminal region/N-lobe of lactoferrin, supporting the biological plausibility of the identified candidates.

Structural analysis: A short sequence (5 amino acids) with a high content of phenylalanine (F) and cysteine (C) suggests high hydrophobicity and the ability to form disulfide bonds (dimerization), which often enhances the antimicrobial activity of short peptides. Belonging to Class 1 confirms its optimal hydrophobicity profile.

SEQ48 showed high activity in the AMPScanner model (0.91) and the SVM model (0.62) but was rejected by the ANN model (0.43). This makes it an interesting “second-tier” candidate whose activity may depend on specific environmental conditions or the target microorganism.

#### 3.1.5. Novelty Check

To confirm the uniqueness of the selected sequences and to exclude previously reported molecules, we performed a targeted screening of the lead peptides using curated international antimicrobial peptide repositories. Specifically, we searched APD3 (Antimicrobial Peptide Database), which compiles annotated AMP sequences from the literature, including natural peptides and documented synthetic analogs, and provides sequence-based search tools for rapid identification of known entries. Within APD3, we carried out an exact-match sequence search to detect identical sequences among catalogued AMPs.

In parallel, we queried DBAASP v3 (Database of Antimicrobial Activity and Structure of Peptides), a manually curated resource focused on peptides with experimentally validated antimicrobial and related cytotoxic/hemolytic) activity. For each peptide, DBAASP includes sequence information, modifications, activity values, and, when available, structural annotations. Within DBAASP, we performed both an exact-match search and a broader screen for homologous sequences among peptides with experimentally confirmed activity, to ensure that the prioritized candidates had not been previously reported under alternative names or as closely related variants.

In this work, the absence of any identical (exact) sequence matches in both repositories (0 hits) was used as an operational criterion supporting scientific novelty at the sequence level for the identified candidates. Notably, for the lead peptide SEQ14 (FCLFK), the searches returned no identical entries (0 hits) in either APD3 or DBAASP at the time of analysis (March 2026). This finding supports classifying SEQ14 as a previously unreported lactoferrin-derived cryptic peptide with potential antimicrobial activity, arising from proteolytic release of short bioactive fragments. The BLASTp analysis confirmed that while these sequences are identical to fragments within the primary structure of equine lactoferrin, they have no high identity matches with previously documented independent antimicrobial peptides, supporting their status as potentially novel cryptic sequences.

### 3.2. Effect of Enzymatic Hydrolysis on In Vitro Antimicrobial Activity

Antimicrobial activity of native lactoferrin and its trypsin hydrolysates was experimentally evaluated using the agar diffusion method against *Escherichia coli*, *Pseudomonas aeruginosa*, and *Staphylococcus aureus* to verify the results of computer-based prediction of hidden antimicrobial peptides in equine lactoferrin.

**Figure 1:**
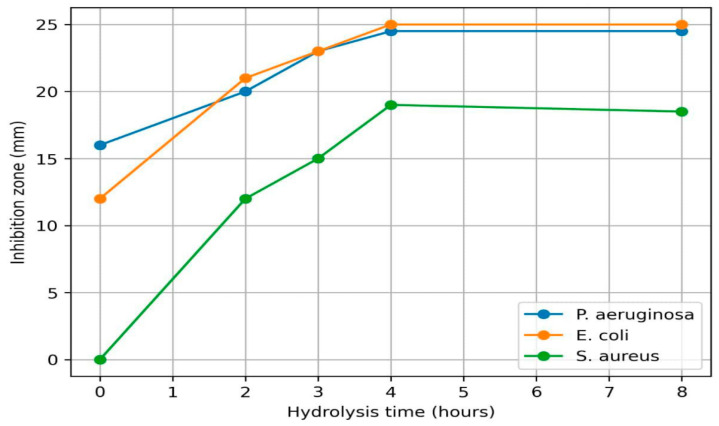
Antimicrobial activity of lactoferrin hydrolysate depending on the hydrolysis time.

The antimicrobial activity increased after enzymatic hydrolysis (Table 3). The diameter of the inhibition zones increased after 2–4 h of hydrolysis, reaching maximum values at 4 h: for *P. aeruginosa*, from 16 mm for native lactoferrin to 24.5 mm after 4 h of hydrolysis; for *E. coli*, from 12 mm to 25 mm; and for *S. aureus*, no activity was observed for native lactoferrin, whereas an inhibition zone of 19 mm was detected after 4 h of hydrolysis. For all three bacterial strains, the 4 h hydrolysate showed significantly larger inhibition zones than native lactoferrin (*p* < 0.05, one-way ANOVA followed by Fisher’s LSD test). These findings indicate that trypsin hydrolysis enhanced the antimicrobial activity of lactoferrin, with the strongest effect observed after 4 h. The peak in antimicrobial activity observed at 4 h of hydrolysis indeed suggests a relatively narrow window for optimal peptide generation. While this study identified this time point as optimal under the tested conditions, we agree that further optimization of hydrolysis parameters (e.g., enzyme concentration, temperature, and pH) could enhance peptide yield and activity. After 8 h of hydrolysis, the activity slightly decreased or stabilized, indicating partial degradation of the active peptide fragments. These data indicate that proteolytic cleavage of lactoferrin leads to the formation of bioactive peptide fragments responsible for the enhancement of antimicrobial activity (Figure 1).

The antimicrobial activity of lactoferrin hydrolysates directly depends on the duration of enzymatic hydrolysis. At the initial stages of hydrolysis, the activity remained relatively low, which may be because proteolytic enzymes only partially cleaved the native protein, and the key cryptic antimicrobial sequences had not yet been released. As the hydrolysis time increased, a noticeable increase in activity was observed, indicating the gradual release of functional peptides with pronounced antimicrobial properties (Table 3).

## 4. Discussion

Targeted enzymatic hydrolysis of equine milk lactoferrin substantially modified its biological properties, transforming the molecule from a predominantly bacteriostatic agent into a powerful source of highly active antimicrobial peptides (AMPs) as it was observed in other species [21]. Interestingly, after reaching a maximum, the activity stabilized or slightly decreased during prolonged hydrolysis. This may reflect the degradation of active peptides or the formation of less active fragments because of excessive protein cleavage. Such patterns are consistent with data from other studies, where the antimicrobial activity of protein hydrolysates is observed within a narrow time window determined by the balance between the formation of functional fragments and their subsequent degradation [22].

Previous studies have shown that enzymatic hydrolysis of lactoferrin can produce bioactive peptides, such as lactoferricin and lactoferrampin, and that their antimicrobial activity depends strongly on the hydrolysis conditions [23,24,25]. The obtained data underscore the importance of optimizing the duration of enzymatic hydrolysis when preparing lactoferrin hydrolysates for use as a source of antimicrobial peptides. Moreover, the dependence of activity on the hydrolysis time can serve as an indicator of the efficiency of the enzymatic process and guide further research aimed at identifying specific cryptic peptide sequences responsible for the antimicrobial effect.

LC–MS/MS identified a diverse range of peptides in the hydrolysates that may contribute to the observed antimicrobial activity [26,27]. Although the activity was measured at the level of the whole hydrolysate, it may result from synergistic interactions among multiple peptides. Suggesting the potential roles of individual sequences will require further studies using isolated or synthetic peptides combined with quantitative assays such as MIC determination. In the present study, we applied a comprehensive approach that ensured strong coherence between the theoretical and experimental stages. Specifically, trypsin specificity was used both in bioinformatic mapping (LC-MS/MS and Mascot) and in macroscopic in vitro validation. This alignment made it possible to correctly match the in silico predictions with the actual dynamics of bioactive fragment accumulation. To prioritize candidate peptides, we adopted a consensus strategy that integrates classical machine learning algorithms (SVM, RF, ANN) with a deep neural network (AMPScanner v.2). This combined approach mitigated the bias of individual methods and yielded a pool of 56 sequences, from which a clear leading peptide emerged, the novel sequence SEQ14 (FCLFK). Notably, the divergence between outputs of classical ML and deep learning tools (AMPScanner v2) highlights the limitations of relying solely on computational predictions. This observation supports the use of the ‘funnel-shaped’ filtering strategy employed here, which allowed us to identify potential leading peptides such as SEQ14 while excluding potential false positives that purely composition-based models might otherwise retain. A short fragment of this candidate, enriched with phenylalanine and cysteine, fully satisfies established physicochemical criteria for membrane-active AMPs, exhibiting high hydrophobicity (Class 1) and a strong propensity to form amphipathic structures. It is important to note that the current in vitro validation was conducted using a total tryptic hydrolysate rather than purified individual peptides. As a result, the antimicrobial activity cannot be definitively attributed to a single sequence, such as SEQ14, as other fragments or synergistic effects within the mixture may contribute to the overall effect. Nevertheless, the peak of antimicrobial activity at 4 h of hydrolysis correlates with the predicted release of these bioactive fragments, providing a successful proof of concept for our multi-stage screening pipeline. It should be emphasized that the observed antimicrobial effect pertains to the general pool of tryptic hydrolysates, providing a proof-of-concept for our screening pipeline rather than direct validation of purified peptides. This approach substantially narrows the search for promising candidates for subsequent chemical synthesis and detailed pharmacological evaluation.

The experimental dependence of antimicrobial activity on the duration of trypsin hydrolysis clearly reflects the classical dynamics of bioactive fragment release. Maximum inhibition is observed at the 4th hour of incubation (up to 25 mm for *E. coli*), indicating that an optimal concentration of medium-chain cationic fragments has been reached, fragments that can effectively interact with bacterial membranes. The subsequent decrease or stabilization of activity (at 8 h) suggests a phase of excessive degradation, during which the predicted AMPs are cleaved into shorter, thermodynamically inactive di- and tripeptides or free amino acids. Additionally, future work will focus on isolating and characterizing the specific bioactive peptides responsible for the observed antimicrobial effects to better understand their structure-function relationships.

It should be noted that a total hydrolysate containing a mixture of peptide fragments was tested, which does not allow the observed activity to be unambiguously attributed to a specific sequence. The enhanced activity against both Gram-negative and Gram-positive bacteria suggests that membrane-active cationic peptides are released during enzymatic cleavage [6,28]. These results confirm the effectiveness of the predictive computational approach used in this study.

An important observation was the expansion of the hydrolysate’s activity spectrum: unlike native lactoferrin, the trypsin pool demonstrated pronounced activity against Gram-positive bacteria (*S. aureus*). The emergence of this activity directly correlates with the results of our in silico analysis, which predicted the release of highly hydrophobic cationic fragments capable of penetrating the peptidoglycan layer and destabilizing the cytoplasmic membrane of Gram-positive pathogens. Similar mechanisms have been described for lactoferrin-derived antimicrobial peptides, which exhibit enhanced activity following enzymatic hydrolysis [29]. In future studies, we will expand the panel of microorganisms to include such strains, allowing us to evaluate the efficacy of the peptides in clinically relevant conditions and gain insight into their potential as alternative or complementary antimicrobials.

Several limitations of the present study should be acknowledged. First, the agar well diffusion method was utilized as a primary screening tool to validate bioinformatics predictions and identify the antimicrobial potential of equine milk lactoferrin hydrolysates. While this approach provided clear qualitative evidence of activity, the absence of Minimum Inhibitory Concentration (MIC) data at this stage limits the ability to make precise quantitative comparisons of individual peptides with standard antibiotics.

Second, the study utilized bacterial strains from a local clinical collection. Although these strains demonstrated stable morphological and biochemical characteristics and are representative of the regional epidemiological context, the importance of international standardization is recognized. Future research will focus on the chemical synthesis of lead peptides (e.g., SEQ14) and their rigorous validation using international reference strains (ATCC) and the broth microdilution method to determine exact MIC values and comprehensive pharmacological profiles.

## 5. Conclusions

At this stage, in vitro testing was carried out on a general pool of trypsin hydrolysates using the agar diffusion method. It should be emphasized that the observed antimicrobial effect pertains to the general pool of tryptic hydrolysates, providing a proof-of-concept for our screening pipeline rather than direct validation of purified peptides. This is a standard approach for primary screening; however, it does not allow the observed bactericidal effect to be unequivocally attributed solely to the isolated SEQ14 sequence.

The current results should be regarded as successful proof of concept, demonstrating the potential effectiveness of the proposed funnel-shaped ML/DL model in identifying hidden AMPs in complex protein matrices.

Our subsequent studies, aimed at conclusively confirming the therapeutic potential of the predicted molecules, will focus on the solid-phase chemical synthesis of peptide SEQ14 and other priority candidates. Follow-up evaluation will include determining the minimum inhibitory concentration (MIC) by the broth microdilution method, investigating bacterial kill kinetics (time-kill assays), and assessing the hemolytic and cytotoxic activity of the purified peptides in vitro. To fully validate the specific contribution of the identified lead peptides, our future research will prioritize the solid-phase synthesis of SEQ14 and other top candidates. This will enable precise determination of MIC values and a deeper understanding of their individual mechanisms of action.

## Figures and Tables

**Table 1:** Consensus predicting matrix for key peptides.

Peptide ID	Sequences	ClassANOVA	RF (Prob)	SVM (Prob)	ANN (Prob)	DL (AMPScanner v2)	Consensus Status
SEQ14	FCLFK	Class 1	0.54	0.67	0.99	0.86	High Confidence (4/4)
SEQ48	LCAGTEADKCACSSQEPYFGYSGAFK	n/a	0.50	0.62	0.43	0.91	Moderate (3/4)
SEQ4	CACSSQEPYFGYSGAFK	Class 1	0.49	0.67	0.54	0.69	Moderate (3/4)
SEQ17	GSGFQLNQLQGVK	Class 2	0.53	0.98	0.91	0.03	False Positive (SVM/ANN bias)
SEQ3	AVANFFSASCVPCADGK	n/a	0.66	0.82	0.71	0.04	False Positive (SVM/ANN bias)
SEQ1	ADAVTLDGGLVYEAGLHPYK	Class 2	0.42	0.52	0.43	0.00	Non-AMP

**Table 3:** Antimicrobial activity of native lactoferrin and its trypsin hydrolysates.

Strain	Sample	Hydrolysis Time (h)	Zone of Inhibition (mm)	Notes/Comparative Analysis
*P. aeruginosa*	Native LF	0	16	Baseline (native LF)
	Hydrolysate	2	20	↑ vs. native LF (*p* < 0.05)
	Hydrolysate	3	23	↑ vs. native LF (*p* < 0.05)
	Hydrolysate	4	24.5	Maximum activity (*p* < 0.05)
	Hydrolysate	8	23.9	Slight decrease vs. 4 h
	Ciprofloxacin	-	31.5	Positive control
*E. coli*	Native LF	0	12	Baseline (native LF)
	Hydrolysate	2	21	↑ vs. native LF (*p* < 0.05)
	Hydrolysate	3	23	↑ vs. native LF (*p* < 0.05)
	Hydrolysate	4	25	Maximum activity (*p* < 0.05)
	Hydrolysate	8	24.7	Slight decrease vs. 4 h
	Ciprofloxacin	-	30.0	Positive control
*S. aureus*	Native LF	0	–	No activity observed
	Hydrolysate	2	12	↑ vs. native LF
	Hydrolysate	3	15	↑ vs. native LF
	Hydrolysate	4	19	Maximum activity
	Hydrolysate	8	18.3	Slight decrease vs. 4 h
	Ciprofloxacin	-	31.0	Positive control

For each bacterial strain, the 4 h hydrolysate showed a significant increase in inhibition zone diameter compared with native lactoferrin (*p* < 0.05).

## Data Availability

The data presented in this study are available in the article and Supplementary Materials. Additional data are available from the corresponding author upon reasonable request.

## Supplementary Materials

The following supporting information can be downloaded at: https://www.mdpi.com/article/10.3390/ani16081223/s1, Table S1: Peptide sequences identified from equine lactoferrin by LC-MS/MS analysis; Table S2: Physicochemical characteristics and predicted bioactivity scores of LC-MS/MS-identified peptides derived from equine lactoferrin.

## Abbreviations

The following abbreviations are used in this manuscript:

AMP	Antimicrobial peptides
AMR	Antimicrobial resistance
ANN	Artificial Neural Network
APD3	Antimicrobial Peptide Database
DBAASP	Database of Antimicrobial Activity and Structure of peptides
GDP	Gross domestic product
eLF	Equine Lactoferrin
MIC	Minimum inhibitory concentration
MS	Molecular weight
pI	Isoelectric point
RF	Random Forest method
SVM	Support vector Machine
