# Supplementary material for: Multifunctional Peptides from Equine Milk Lactoferrin: Evaluation of Antimicrobial Activity In Silico and In Vitro
Source: Animals (Basel). 2026 Apr 16;16(8):1223. doi: 10.3390/ani16081223 (PMC13113733; doi:10.3390/ani16081223)
Supplement: Supplementary file 1 [file animals-16-01223-s001.zip › animals-4226350-supplementary.pdf]

Supplementary Materials S1

Supplementary Table S1. Peptide sequences identified from equine lactoferrin by LC-MS/MS analysis

№	Peptide	Start	End	Length
1	R.WCTISPAEAAK.C	14	24	11
2	R.GPSVSCIR.K	37	44	8
3	R.KTSSFECIQAIAANK.A	45	59	15
4	K.TSSFECIQAIAANK.A	46	59	14
5	K.ADAVTLDGGLVYEAGLHPYK.L	60	79	20
6	K.LRPVAAEVYQTR.G	80	91	12
7	R.YYAVAVVK.K	98	105	8
8	K.KGSGFQLNQLQGVK.S	106	119	14
9	K.GSGFQLNQLQGVK.S	107	119	13
10	K.AVANFFSASCVPCADGK.Q	154	170	17
11	K.QYPNLCR.L	171	177	7
12	R.LCAGTEADKCACSSQEPYFGYSGAFK.C	178	203	26
13	K.CACSSQEPYFGYSGAFK.C	187	203	17
14	K.CLENGAGDVAFVK.D	204	216	13
15	K.DSTVFENLPDEADR.D	217	230	14
16	K.DSTVFENLPDEADRDK.Y	217	232	16
17	K.DSTVFENLPDEADRDKYELLCPDNTR.K	217	242	26
18	K.YELLCPDNTR.K	233	242	10
19	R.VPSHAVVAR.S	256	264	9
20	R.SVDGREDLIWR.L	265	275	11
21	R.EDLIWR.L	270	275	6
22	K.SSAFQLFK.S	289	296	8
23	K.STPENKDLLFK.D	297	307	11
24	K.DLLFKDSALGFVR.I	303	315	13
25	K.DSALGFVR.I	308	315	8
26	R.IPSQIDSGLYLGANYLTATQNLR.E	316	338	23
27	R.ETAAEVAAR.R	339	347	9
28	R.VVWCAVGPEEER.K	351	362	12
29	R.VVWCAVGPEEERK.C	351	363	13
30	K.QWSDVSNR.K	366	373	8
31	R.KVACASASTTEECIALVLK.G	374	392	19
32	K.VACASASTTEECIALVLK.G	375	392	18
33	K.VACASASTTEECIALVLKGEADALNLDGGFIYVAGK.C	375	410	36
34	K.GEADALNLDGGFIYVAGK.C	393	410	18
35	K.CGLVPVLAENQK.S	411	422	12
36	K.SQNSNAPDCVHRPPEGYLAVAVVR.K	423	446	24
37	R.KSDADLTWNSLSGK.K	447	460	14
38	R.KSDADLTWNSLSGKK.S	447	461	15
39	K.SDADLTWNSLSGK.K	448	460	13
40	K.FFSQSCAPGADPQSSLCALCVGNNENENK.C	492	520	29
41	R.YYGYTGAFR.C	529	537	9
42	K.AGDVAFVK.D	543	550	8
43	K.DVTVLQNTDGK.N	551	561	11

44	K.NSEPWAK.D	562	568	7
45	K.DLKQEDFELLCLDGTR.K	569	584	16
46	K.QEDFELLCLDGTR.K	572	584	13
47	R.KPVAEAESCHLAR.A	585	597	13
48	R.APNHAVVSQSDR.A	598	609	12
49	K.KVLFLQQDQFGGNGPDCPGK.F	615	634	20
50	K.VLFLQQDQFGGNGPDCPGK.F	616	634	19
51	K.FCLFK.S	635	639	5
52	K.NLLFNDNTECLAELQGK.T	644	660	17
53	K.TTYEQYLGSEYVTSITNLR.R	661	679	19
54	K.TTYEQYLGSEYVTSITNLRR.C	661	680	20
55	R.RCSSSPLLEACAFLR.A	680	694	15
56	R.CSSSPLLEACAFLR.A	681	694	14

Supplementary Materials S2

Supplementary Table S2. Physicochemical characteristics and predicted bioactivity scores of LC-MS/MS-identified peptides derived from equine lactoferrin

Peptide	Score-Bioact	MS	pI	Aliphatic	Gravy	Boman	Classe
FCLFK	0,95	713,35	8,22	78,00	1,60	-1,32	1
CSSSPLLEACAFLR	0,91	1609,77	5,99	97,86	0,77	0,57	1
RCSSSPLLEACAFLR	0,86	1765,86	8,07	91,33	0,42	1,52	1
SSAFQLFK	0,85	926,48	8,47	61,25	0,28	0,65	1
CACSSQEPYFGYSGAFK	0,76	1957,80	5,99	11,76	-0,24	0,84	2
QYPNLCR	0,75	949,44	8,22	55,71	-1,16	3,00	2
AVANFFSASCVPCADGK	0,69	1799,80	5,86	57,65	0,69	0,17	1
IPSQIDSGLYLGANYLTATQNLR	0,69	2507,32	5,83	110,43	-0,10	1,09	1
DLLFKDSALGFVR	0,66	1479,80	5,96	120,00	0,49	1,06	1
YYGYTGAFR	0,62	1096,50	8,50	11,11	-0,59	1,24	2
DSALGFVR	0,62	863,45	5,84	97,50	0,43	1,54	1
GPSVSCIR	0,60	874,43	8,25	85,00	0,39	1,31	1
KSDADLTWNSLSGKK	0,59	1648,85	8,50	58,67	-1,15	2,57	2
DSTVFENLPDEADRDKYELLCPDNTR	0,58	3111,42	4,03	60,00	-1,23	3,71	2
LCAGTEADKCACSSQEPYFGYSGAFK	0,55	2903,23	4,68	30,38	-0,24	1,04	2
EDLIWR	0,54	830,43	4,37	130,00	-0,68	3,04	2
GSGFQLNQLQGVK	0,53	1374,73	10,12	82,31	-0,41	0,96	2
KVLFLQQDQFGGNGPDCPGK	0,53	2204,08	5,95	53,50	-0,69	1,34	2
ADAVTLDGGLVYEAGLHPYK	0,52	2088,05	4,54	102,50	0,08	0,31	1
VLFLQQDQFGGNGPDCPGK	0,52	2075,99	4,21	56,32	-0,52	1,12	2
QEDFELLCLDGTR	0,52	1594,74	3,92	90,00	-0,49	2,62	2
GEADALNLDGGFIYVAGK	0,52	1808,90	4,03	97,78	0,21	0,31	1
DLKQEDFELLCLDGTR	0,51	1950,94	4,11	97,50	-0,63	2,71	2
FFSQSCAPGADPQSSLCALCVGNNENE NK	0,50	3200,37	4,14	47,24	-0,42	1,65	2
KSDADLTWNSLSGK	0,50	1520,76	5,96	62,86	-0,95	2,35	2

SDADLTWNSLSGK	0,50	1392,66	4,21	67,69	-0,72	2,11	2
KGSGFQLNQLQGVK	0,47	1502,82	10,00	76,43	-0,66	1,29	2
NSEPWAK	0,47	830,39	6,00	14,29	-1,77	2,60	2
STPENKDLLFK	0,42	1290,68	5,79	70,91	-1,00	2,40	2
KPVAEAESCHLAR	0,41	1466,74	6,75	75,38	-0,39	2,03	2
QWSDVSNR	0,41	990,45	5,84	36,25	-1,66	4,53	2
AGDVAFVK	0,38	805,43	5,88	97,50	0,88	-0,16	1
CLENGAGDVAFVK	0,37	1378,66	4,37	90,00	0,45	0,38	1
SVDGREDLIWR	0,37	1344,69	4,56	97,27	-0,83	3,66	2
WCTISPAEAAK	0,30	1232,59	5,99	62,73	0,09	0,39	2
CGLVPVLAENQK	0,29	1326,69	5,99	121,67	0,33	0,21	1
VACASASTTEECIALVLK	0,25	1921,96	4,53	119,44	1,04	-0,08	1
VVWCAVGPEEERK	0,25	1557,77	4,79	74,62	-0,38	1,72	2
KTSSFECIQAIAANK	0,24	1666,84	8,20	72,00	-0,06	1,32	2
VPSHAVVAR	0,24	934,53	9,73	118,89	0,68	0,80	1
SQNSNAPDCVHRPPEGYLAVAVVR	0,24	2635,30	6,47	77,08	-0,39	1,96	2
TSSFECIQAIAANK	0,23	1538,74	5,66	77,14	0,21	1,02	2
NLLFNDNTECLAELQGK	0,23	1977,95	4,14	97,65	-0,42	1,71	2
KVACASASTTEECIALVLK	0,23	2050,05	6,13	113,16	0,78	0,21	1
VVWCAVGPEEER	0,20	1429,67	4,25	80,83	-0,08	1,40	2
YELLCPDNTR	0,20	1279,60	4,37	78,00	-0,85	2,86	2
LRPVAAEVYQTR	0,13	1401,77	8,75	97,50	-0,32	2,35	2
APNHAVVSQSDR	0,12	1279,63	6,79	65,00	-0,78	2,96	2
VACASASTTEECIALVLKGEADALNLD GGFIYVAGK	0,12	3712,86	4,18	108,61	0,63	0,11	1
DSTVFENLPDEADRDK	0,11	1849,84	3,96	48,75	-1,46	4,23	2
DSTVFENLPDEADR	0,10	1606,72	3,77	55,71	-1,14	3,82	2
YYAVAVVK	0,09	911,51	8,50	133,75	1,21	-1,23	1
DVTVLQNTDGK	0,08	1188,60	4,21	88,18	-0,68	2,39	2
TTYEQYLGSEYVTSITNLR	0,07	2237,10	4,53	76,84	-0,57	2,02	2
TTYEQYLGSEYVTSITNLRR	0,07	2393,20	5,81	73,00	-0,77	2,67	2
ETAAEVAAR	0,06	916,46	4,53	76,67	-0,09	2,20	2
